# Solitary Fibrous Tumor of the Greater Omentum with Intratumoral Infarction: A Case Report

**DOI:** 10.70352/scrj.cr.26-0098

**Published:** 2026-05-29

**Authors:** Takumi Funo, Hidetaka Kawamura, Yukitoshi Todate, Noriyuki Uesugi, Toshiyuki Saginoya, Nobuyasu Suzuki, Tamotsu Sugai, Michitaka Honda

**Affiliations:** 1 Department of Surgery, Southern TOHOKU General Hospital, Koriyama, Fukushima, Japan; 2 Department of Minimally Invasive Surgical and Medical Oncology, Fukushima Medical University, Koriyama, Fukushima, Japan; 3 Department of Pathology, Southern TOHOKU General Hospital, Koriyama, Fukushima, Japan; 4 Department of Diagnostic Radiology, Southern TOHOKU General Hospital, Koriyama, Fukushima, Japan

**Keywords:** solitary fibrous tumor, greater omentum, omental tumor, intratumoral infarction, abdominal pain, MRI

## Abstract

**INTRODUCTION:**

Solitary fibrous tumors (SFTs) are rare mesenchymal tumors with rich vascularity that rarely arise from the greater omentum. Many cases of SFT are asymptomatic and discovered incidentally; however, symptomatic cases may occur when complications such as intratumoral infarction develop. We report a case of an SFT originating from the greater omentum, complicated by intratumoral infarction.

**CASE PRESENTATION:**

A 71-year-old woman presented to the emergency department with a 2-day history of worsening abdominal pain. Laboratory testing revealed elevated inflammatory markers, with a white blood cell count of 17.0 × 10^3^/μL and C-reactive protein level of 11.34 mg/dL. Contrast-enhanced CT demonstrated an approximately 80-mm, relatively homogeneous abdominal mass with poor internal enhancement. MRI showed uniformly low signal intensity on T1-weighted images and a heterogeneous mixture of intermediate and low signal intensity on T2-weighted images. Diffusion-weighted imaging revealed hyperintensity corresponding to the intermediate T2-signal area, whereas the T2-low-signal regions remained hypointense. Conservative management was initiated, resulting in a gradual improvement of abdominal pain and inflammatory markers. On hospital day 8, laparotomy was performed, and the tumor, arising from and continuous with the greater omentum, was completely resected. Pathology demonstrated intratumoral infarction, and immunohistochemical findings confirmed the diagnosis of an SFT. The postoperative course was uneventful, and no recurrence was observed.

**CONCLUSIONS:**

SFTs arising from the greater omentum may develop intratumoral infarction, presenting with acute abdominal pain and inflammatory responses. Cross-sectional imaging modalities, such as CT and MRI, can assist in identifying this uncommon pathological condition.

## Abbreviations

α-SMA
: α-smooth muscle actin
CRP
: C-reactive protein
DWI
: diffusion-weighted imaging
GIST
: gastrointestinal stromal tumors
PgR
: progesterone receptor
SFT
: solitary fibrous tumor
WBC
: white blood cell

## INTRODUCTION

SFTs are rare mesenchymal neoplasms that can arise in various anatomical locations, including the thoracic cavity, head and neck, retroperitoneum, and abdominal cavity.^1–3)^ Histologically, SFTs exhibit a characteristic patternless architecture with a variable collagenous stroma.^4)^ Most tumors show strong nuclear signal transducer and activator of transcription 6 (STAT6) expression, which reflects *NAB2*–*STAT6* gene fusion and serves as a highly specific diagnostic hallmark.^5,6)^

Many SFTs are asymptomatic and discovered incidentally; however, a subset demonstrates malignant potential, including local recurrence and distant metastasis.^7,8)^ Therefore, complete surgical resection with adequate margins is essential for achieving long-term disease control.^7)^ Consequently, preoperative recognition of SFT based on clinical and radiological features is important for planning an appropriate surgical strategy.

Extrapleural SFTs are increasingly recognized; however, those arising from the greater omentum are exceedingly rare.^9)^ Owing to their rich vascularity, SFTs typically appear as well-enhanced masses on contrast-enhanced CT and exhibit variable MRI features, depending on their collagen and vascular composition.^8,10)^ However, intratumoral infarction, although uncommon, may significantly alter the imaging characteristics and lead to acute abdominal pain and inflammatory responses, making preoperative diagnosis challenging.

Here, we report a rare case of an SFT of the greater omentum complicated by intratumoral infarction. We describe the clinical and radiological findings and review the relevant literature to highlight the diagnostic considerations for this uncommon presentation.

## CASE PRESENTATION

A 71-year-old woman presented to the emergency department with a 2-day history of worsening abdominal pain. On admission, her vital signs were as follows: body temperature, 37.0°C; blood pressure, 180/101 mmHg; and pulse rate, 92 beats/min. Physical examination revealed a flat and soft abdomen with tenderness localized to the left lower abdominal quadrant without muscular guarding. Laboratory testing showed a marked inflammatory response, with an elevated WBC count of 17.0 × 10^3^/μL and CRP level of 11.34 mg/dL.

**Fig. 1:**
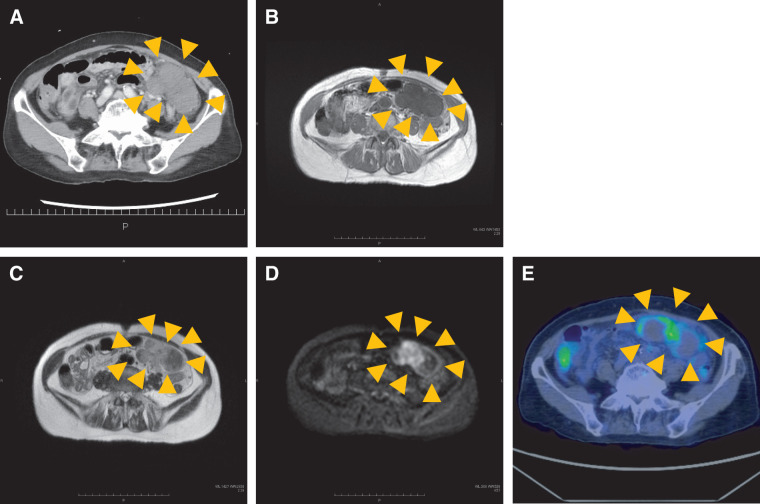
Preoperative radiologic findings. (**A**) Contrast-enhanced CT at presentation demonstrates an approximately 80-mm, relatively homogeneous mass in the left abdomen with poor internal enhancement. (**B**) T1-weighted image shows uniformly low signal intensity within the tumor. (**C**) T2-weighted image demonstrates heterogeneous signal intensity, consisting of intermediate-signal areas and distinct low-signal regions. (**D**) DWI shows high signal intensity in the intermediate T2-signal areas, whereas the T2-low-signal regions appear hypointense. (**E**) PET-CT shows SUVmax: 4.6 accumulation along the edge of the tumorous lesion, with no abnormal uptake elsewhere. DWI, diffusion-weighted imaging; SUVmax, maximum standardized uptake value

Contrast-enhanced CT at presentation revealed an approximately 80-mm, relatively homogeneous, multinodular mass in the left abdomen with poor internal enhancement (**Fig. 1A**). The mass showed no continuity with the gastrointestinal tract, and there were no signs of bowel obstruction or metastasis. Based on these findings, an omental tumor was suspected. Given the presence of acute abdominal pain, elevated inflammatory markers, and poor internal enhancement of the lesion, intratumoral infection was initially considered.

The patient was admitted on the same day and managed conservatively with nil per os, intravenous fluid supplementation, and intravenous piperacillin/tazobactam (4.5 g every 6 h). By the second day of hospitalization, her abdominal pain had improved, and inflammatory markers had decreased (WBC, 8.4 × 10^3^/μL; CRP, 6.67 mg/dL).

To further characterize the lesion and reassess the diagnosis while monitoring the response to antibiotics, additional imaging studies, including MRI and PET-CT, were performed.

MRI performed on hospital day 3 demonstrated uniformly low signal intensities on T1-weighted images (**Fig. 1B**) and a heterogeneous mixture of intermediate and low signal intensities on T2-weighted images (**Fig. 1C**). On DWI, the areas showing intermediate T2 signal intensity demonstrated high signal intensity, whereas the T2-low-signal regions remained hypointense (**Fig. 1D**). PET-CT on hospital day 3 showed mild to moderate uptake (maximum standardized uptake value: 4.6) localized to the peripheral region of the tumor, with no abnormal uptake elsewhere (**Fig. 1E**).

Based on these imaging findings, the differential diagnosis included GISTs, desmoid tumor, and malignant lymphoma. Although SFT was also considered, it was not strongly suspected preoperatively because the lesion lacked the typical hypervascular enhancement on contrast-enhanced CT and showed atypical imaging features possibly related to secondary changes such as intratumoral infarction.

**Fig. 2:**
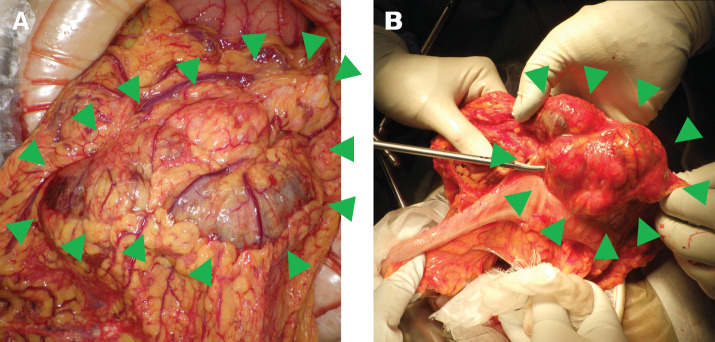
Intraoperative findings. (**A**) Laparotomy reveals a multinodular mass arising from the greater omentum. (**B**) The tumor is shown in close proximity to the transverse colon during resection, without direct invasion.

As a definitive diagnosis could not be established preoperatively, surgical resection was performed on hospital day 8 for both diagnostic confirmation and therapeutic purposes. Midline laparotomy revealed a multinodular mass arising from the greater omentum along the right gastroepiploic vessels, near the midportion of the stomach (**Fig. 2A** and **2B**). No direct invasion of adjacent organs was observed. The greater omentum containing the mass was mobilized, and the right gastroepiploic artery and vein were ligated and divided near their origins. The tumor was completely resected with adequate margins. The operative duration was 87 min, and the estimated blood loss was 20 mL.

**Fig. 3:**
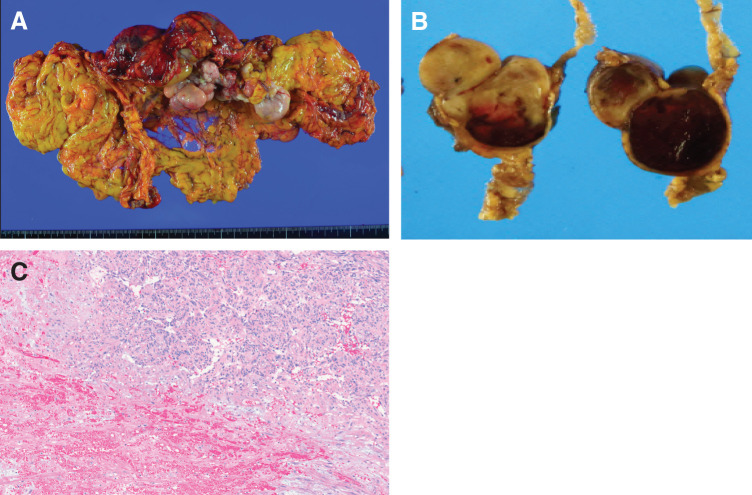
Gross and histopathological findings of the resected specimen. (**A**) Gross examination of the excised greater omentum shows multiple nodular lesions of varying sizes, measuring up to 130 × 65 mm. (**B**) On cut surface, viable tumor areas appear whitish, whereas infarcted regions are dark red, with areas of hemorrhage and hyalinization. (**C**) Hematoxylin and eosin–stained section demonstrates a spindle cell proliferation with low to moderate cellularity and mild nuclear atypia. Areas of infarction, hyalinization, and hemorrhage are observed, and no mitotic figures are identified.

**Fig. 4:**
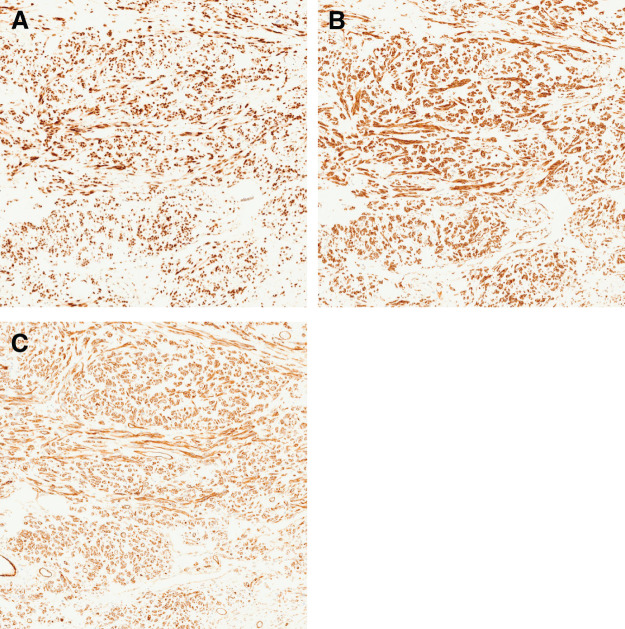
Immunohistochemical findings. (**A**) Immunohistochemical staining for STAT6 shows diffuse strong nuclear positivity in tumor cells. (**B**) Tumor cells exhibit diffuse cytoplasmic positivity for BCL-2. (**C**) CD34 immunostaining highlights the rich vascular network and diffuse membranous/cytoplasmic positivity in tumor cells, supporting the diagnosis of SFT. SFT, solitary fibrous tumor; STAT6, signal transducer and activator of transcription 6

Histopathological examination of the resected specimen revealed multiple nodular lesions of varying sizes, measuring up to 130 × 65 mm, within the excised greater omentum (**Fig. 3A**). The tumor exhibited areas of infarction, hyalinization, and hemorrhage without tumor necrosis, along with low- to moderate-density proliferation of spindle cells displaying mild atypia; however, no mitotic figures were identified (**Fig. 3B** and **3C**). These findings support the diagnosis of infarction rather than necrosis, based on the preservation of tissue architecture. Immunohistochemical analysis revealed strong positivity for STAT6, BCL-2, CD34, and vimentin; partial positivity for desmin; and weak nuclear staining for PgR (**Fig. 4A**–**4C**). The tumor was negative for β-catenin (nuclear), S-100, α-SMA, and KIT. These findings were consistent with the diagnosis of a primary SFT of the greater omentum.

Oral water intake was initiated on POD 1, and a soft diet was introduced on POD 2. The postoperative course was uneventful, and the patient was discharged on POD 10. Six months postoperatively, the patient remained under surveillance with routine blood tests and contrast-enhanced CT, with no evidence of recurrence or metastasis.

## DISCUSSION

We report a rare case of an SFT arising from the greater omentum, in which intratumoral infarction was the primary cause of acute abdominal pain. Although SFTs are usually asymptomatic,^7)^ infarction can lead to acute abdominal pain and inflammatory response. In this case, contrast-enhanced CT demonstrated a relatively homogeneous abdominal mass with poor internal enhancement, whereas MRI revealed a uniformly low signal intensity on T1-weighted images and heterogeneous intermediate-to-low signal intensity on T2-weighted images. DWI revealed hyperintensity in the intermediate T2-signal areas corresponding to viable tumor components, whereas the T2-low-signal regions remained hypointense, consistent with infarcted tissue. These clinical and radiologic features are useful for the differential diagnosis of omental tumors, particularly when infarction obscures the typical hypervascular appearance of SFT.

Primary SFTs of the greater omentum are extremely rare, which contributes to the diagnostic difficulty in clinical practice. We searched PubMed using the keywords “SFT” AND (“greater omentum” OR “omentum”) for articles published from database inception through December 2025. After excluding cases originating from the lesser omentum or gallbladder and those with insufficient clinical details, a total of 24 cases of SFT arising from the greater omentum were identified (**Supplementary Material 1**). As summarized in **Table 1**, the reported patients ranged widely in age (23–85 years), with no clear sex predominance. Approximately half of the reported cases were incidentally detected and asymptomatic (11/24, 46%), whereas 13 patients (54%) were symptomatic. Among symptomatic cases, abdominal pain was the most common presenting symptom (7/13, 54%), followed by abdominal distension or bloating (5/13, 38%), as well as nausea, vomiting, or fever.

**Table 1:** Summary of cases with SFT originating from the greater omentum^a^

Author (year)	Age/sex	Symptoms	Tumor size (cm)	Necrosis	Pathology	Presumed cause of symptoms	CT/MRI findings
Patriti et al. (2006)^12)^	24/M	Abdominal pain, fever	3.2	Absence	Benign	Tumor hemorrhage	CT: Hemangioma-like tumor with ascites
Salem et al. (2008)	60/M	Abdominal pain	24	Absence	Malignant	Mass effect	CT: Lobulated heterogeneous mass
Garbin et al. (2011)	27/F	Asymptomatic	NA	NA	NA	NA	CT: Hypervascular heterogeneous lesion
Zong et al. (2012)	29/M	Epigastric discomfort, compression	21	Absence	Benign	Mass effect	CT: Homogeneous mass with poor enhancement
Becker et al. (2014)^15)^	41/F	Abdominal bloating, vomiting	30	Absence	Benign	Mass effect	CT: Large vascular heterogeneous mass with multiple cystic and necrotic areas
Osawa et al. (2014)	32/F	Asymptomatic	4.8	Absence	Benign	None	NA
Sato et al. (2014)	85/F	Abdominal bloating	19	Presence	Benign	Mass effect	CT: Large hypervascular mass
Harada et al. (2014)^14)^	62/F	Asymptomatic	10	Absence	Malignant	None	CT: Homogeneous mass with poor internal enhancement MRI: Low T1, heterogeneous high-to-intermediate T2, high DWI
Urabe et al. (2015)	52/M	Asymptomatic	5	Absence	Benign	None	CT: Progressive heterogeneous enhancement MRI: Low T1, slightly high T2
Cazejust et al. (2015)	68/M	Asymptomatic	4.5	Absence	Benign	None	CT: Delayed-phase enhancement
Michiura et al. (2016)	36/M	Asymptomatic	9	Absence	Benign	None	CT: Heterogeneous enhanced mass
Archid et al. (2016)	24/M	Abdominal pain	8	Absence	Benign	Mass effect	CT: Hypervascular inhomogeneous tumor
Rodriguez-Tarrega et al. (2016)	34/F	Asymptomatic	6	Absence	Malignant	None	NA
Moszynski et al. (2016)	29/F	Loss of appetite, abdominal bloating	4	NA	Benign	Mass effect	CT: Polycyclic enhancing structures
Vasdeki et al. (2018)	72/M	Abdominal pain	11	Presence	Malignant	Mass effect	CT: Heterogeneous hypervascular mass
Jung et al. (2019)	57/M	Asymptomatic	10	Absence	Malignant	None	CT: Strongly enhancing mass
Guo et al. (2021)	64/F	Asymptomatic	27	Presence	Malignant	None	CT: Mixed-density heterogeneous mass
Ingle et al. (2021)^11)^	37/F	Abdominal pain	6	Absence	Malignant	Partial torsion	CT: Homogeneous mass
Eltawil et al. (2021)	63/M	Abdominal pain, nausea	14	Presence	Benign	Mass effect	CT: Peripheral enhancement with central hypodensity
Shahid et al. (2021)	61/M	Abdominal bloating	17	Absence	Malignant	Mass effect	CT: Hypodense heterogenous mass
Yin et al. (2022)	23/M	Abdominal pain	9	Absence	NA	Mass effect	CT: Homogeneous mass MRI: Isointense or slightly hyperintense both T1 and T2
Tuan et al. (2022)	31/F	Abdominal bloating	7.6	Absence	Benign	Mass effect	CT: Homogeneous enhancement
Gendvilaitė et al. (2023)	32/M	Asymptomatic	10.5	Presence	Benign	None	MRI: High T1, heterogeneous intermediate-to-low T2
Hatayama et al. (2025)	53/F	Asymptomatic	2.8	Absence	Benign	None	CT: Homogeneous tumor

^a^Relevant articles reporting SFTs originating from the greater omentum were identified and reviewed. Only cases with sufficient clinical information were included in the analysis.

DWI, diffusion-weighted imaging; NA, not available; SFT, solitary fibrous tumor

Symptomatic presentation of greater omental SFT is influenced not only by tumor size but also by secondary intratumoral events. As summarized in **Table 1**, symptomatic presentation of greater omental SFT tends to be associated with larger tumor size, most likely due to mass effect or secondary degenerative changes. Indeed, many symptomatic cases involved tumors larger than 10 cm. However, tumor size alone does not fully explain symptom onset. Several relatively small tumors (approximately 3–6 cm) have also been reported to cause abdominal pain, often due to acute events such as torsion^11)^ or tumor hemorrhage^12)^ rather than simple mass effect. Similarly, intratumoral infarction may arise from various mechanisms, including rapid tumor growth exceeding its blood supply, spontaneous vascular occlusion, or mechanical factors such as torsion of the omental pedicle or vascular compression. In the present case, no macroscopic torsion was identified intraoperatively, suggesting that localized vascular compromise, possibly related to the relatively large tumor size, may have contributed to the development of infarction. These findings indicate that the development of acute symptoms in SFT is more closely related to secondary intratumoral events than to tumor size itself.

In the present case, although the tumor was relatively large (130 mm), the acute abdominal symptoms were unlikely to be explained solely by mass effect. Instead, extensive intratumoral infarction, supported by poor internal enhancement on CT and corresponding pathological findings, likely resulted in a sudden reduction in tumor perfusion and subsequent inflammatory response, leading to acute abdominal pain. Notably, intratumoral infarction has not been clearly described as a primary cause of acute symptoms in previously reported cases of greater omental SFT, suggesting that this mechanism may represent a distinct and clinically important presentation. Furthermore, the clinical course in the present case supports this interpretation. The patient’s abdominal pain and inflammatory response improved with conservative management alone. Histopathologically, infarction was observed without evidence of tumor necrosis, suggesting that the ischemic change was limited and did not progress to irreversible tissue damage. In such cases, infarction may represent a self-limiting process, whereas progression to necrosis is generally associated with persistent inflammation or complications. Although direct evidence in SFT is lacking, primary omental infarction has been reported to resolve with conservative treatment.^13)^ Therefore, the abdominal symptoms in this case were considered to be transient and attributable to localized intratumoral infarction. Because the patient’s symptoms and inflammatory response improved with conservative management, emergency surgery was not required. Therefore, surgical resection was performed electively after clinical stabilization and further imaging evaluation.

In SFTs, heterogeneous signal intensity on T2-weighted MRI generally reflects the admixture of fibrous stroma and cellular tumor components.^8)^ Consistent with this concept, the previously reported case by Harada et al. in **Table 1** demonstrated heterogeneous intermediate-to-high signal intensity on T2-weighted images, findings attributable to variations in collagen content and cellularity.^14)^ On contrast-enhanced CT, SFTs often show prominent enhancement reflecting their rich vascularity^8)^; however, enhancement patterns can be variable, particularly in large tumors with secondary changes such as necrosis or hemorrhage.^12,15)^

In contrast to these typical imaging characteristics, the present case demonstrated atypical multimodal imaging findings attributable to extensive intratumoral infarction. Contrast-enhanced CT revealed a relatively homogeneous mass with poor internal enhancement, indicating markedly reduced intratumoral perfusion. On MRI, infarcted regions showed low signal intensity on both T2-weighted imaging and DWI, whereas viable tumor components exhibited intermediate T2 signal intensity with high signal intensity on DWI, consistent with existing literature.^16,17)^ These findings suggest that intratumoral infarction can obscure the characteristic hypervascular appearance of SFTs on contrast-enhanced CT and substantially alter expected MRI signal characteristics. Accordingly, careful integration of CT and MRI findings with clinical symptoms and inflammatory markers may facilitate recognition of intratumoral infarction as a distinct mechanism underlying acute presentation in greater omental SFTs. In clinical practice, such atypical imaging findings, particularly the absence of characteristic hypervascular enhancement, may broaden the differential diagnosis and lower the preoperative suspicion for SFT, as observed in the present case. Therefore, SFT should be considered even when imaging features are altered by secondary changes such as intratumoral infarction.

A limitation of this study is the relatively short follow-up period of 6 months. Given that SFTs are known to have the potential for late recurrence, long-term follow-up is required.

## CONCLUSIONS

Intratumoral infarction in SFTs of the greater omentum can result in acute abdominal pain accompanied by inflammatory responses. Cross-sectional imaging, particularly contrast-enhanced CT and MRI, plays a crucial role in recognizing this uncommon pathological condition. Awareness of this mechanism may improve diagnostic accuracy and support appropriate clinical management of symptomatic greateromental SFTs.

## SUPPLEMENTARY MATERIAL

Supplementary Material 1

## DECLARATIONS

### Availability of data and materials

All data generated or analyzed during this study are included in this published article and its supplementary information files.
