# Supplementary material for: Solitary Fibrous Tumor of the Greater Omentum with Intratumoral Infarction: A Case Report
Source: Surg Case Rep. 2026 May 29;12(1):26-0098. doi: 10.70352/scrj.cr.26-0098 (PMC13222725; doi:10.70352/scrj.cr.26-0098)
Supplement: Supplementary Material 1 [file scr-12-01-26-0098-s001.pdf]

Supplementary material 1. Chronological list of published cases of solitary fibrous tumors originating from the greater omentum.

Literature search strategy
A literature review was conducted using the PubMed database on December 3, 2025. The search was performed using the following terms applied to the Title/Abstract fields: (“solitary fibrous tumor” OR “solitary fibrous tumour” OR SFT) AND (“omentum” OR “omental” OR “greater omentum”).

1. Layfield LJ, Gopez EV. Percutaneous image-guided fine-needle aspiration of peritoneal lesions. Diagn Cytopathol. 2003 Jan;28(1):6-12. doi: 10.1002/dc.10217.
2. Thalheimer A, Meyer D, Gattenlöhner S, et al. Gastrointestinaler Stromatumor der Bauchwand. Ungewöhnliche Lokalisation eines seltenen Tumors [Gastrointestinal stromal tumor of the abdominal wall. An unusual localization of a rare tumor]. Chirurg. 2004 Jul;75(7):708-12. German. doi: 10.1007/s00104-003-0696-5.
3. Patriti A, Rondelli F, Gullà N, et al. Laparoscopic treatment of a solitary fibrous tumor of the greater omentum presenting as spontaneous haemoperitoneum. Ann Ital Chir. 2006 Jul-Aug;77(4):351-3.
4. Salem AM, Bateson PB, Madden MM. Large solitary fibrous tumor arising from the omentum. Saudi Med J. 2008 Apr;29(4):617-8.
5. Mosquera JM, Fletcher CD. Expanding the spectrum of malignant progression in solitary fibrous tumors: a study of 8 cases with a discrete anaplastic component --is this dedifferentiated SFT? Am J Surg Pathol. 2009 Sep;33(9):1314-21. doi: 10.1097/pas.0b013e3181a6cd33.
6. Ekıcı Y, Uysal S, Güven G, et al. Solitary fibrous tumor of the lesser omentum: report of a rare case. Turk J Gastroenterol. 2010 Dec;21(4):464-6.
7. Garbin O, Hummel M, Diana M, et al. Solitary fibrous tumor of the great omentum. J Minim Invasive Gynecol. 2011 Nov-Dec;18(6):694-5. doi: 10.1016/j.jmig.2011.01.013.
8. Zong L, Chen P, Wang GY, et al. Giant solitary fibrous tumor arising from greater omentum. World J Gastroenterol. 2012 Nov 28;18(44):6515-20. doi: 10.3748/wjg.v18.i44.6515.
9. Osawa H, Nishimura J, Inoue A, et al. [A case of solitary fibrous tumor from the greater omentum resected via laparoscopic surgery]. Gan To Kagaku Ryoho. 2014 Nov;41(12):2493-5. Japanese.
10. Sato T, Yamaguchi S, Koyama I, et al. Acute life-threatening portal venous dilatation induced by a huge solitary fibrous tumor of the omentum. Hepatogastroenterology. 2014 Nov-Dec;61(136):2200-2.
11. Becker JHR, Koto MZ, Matsevych OY, et al. Haemangiopericytoma/solitary fibrous tumour of the greater omentum. S Afr J Surg. 2014 Nov;52(4):111-113. doi: 10.7196/sajs.2274.

12. Harada N, Nobuhara I, Haruta N, et al. Concurrent Malignant Solitary Fibrous Tumor Arising from the Omentum and Grade 3 Endometrial Endometrioid Adenocarcinoma of the Uterus with p53 Immunoreactivity. Case Rep Obstet Gynecol. 2014;2014:216340. doi: 10.1155/2014/216340.
13. Urabe M, Yamagata Y, Aikou S, et al. Solitary fibrous tumor of the greater omentum, mimicking gastrointestinal stromal tumor of the small intestine: a case report. Int Surg. 2015 May;100(5):836-40. doi: 10.9738/INTSURG-D-14-00141.1.
14. Cazejust J, Wendum D, Bourrier A, et al. Solitary fibrous tumor of the greater omentum. Diagn Interv Imaging. 2015 Sep;96(9):959-61. doi: 10.1016/j.diii.2014.12.006.
15. Michiura T, Yamabe K, Hayashi N, et al. [A Surgical Case of Solitary Fibrous Tumor Originating from the Greater Omentum]. Gan To Kagaku Ryoho. 2016 Nov;43(12):2265-67. Japanese.
16. Shetty KJ, Rao C, Prasad HL. Glomangiopericytoma Versus Solitary Fibrous Tumor: an Omental Tumor with Unusual Diagnostic Dilemma. Indian J Surg Oncol. 2016 Dec;7(4):475-478. doi: 10.1007/s13193-016-0522-4.
17. Rodriguez Tarrega E, Hidalgo Mora JJ, Paya Amate V, et al. Solitary fibrous tumor of the greater omentum mimicking an ovarian tumor in a young woman. Gynecol Oncol Rep. 2016 Apr 26;17:16-9. doi: 10.1016/j.gore.2016.04.004.
18. Archid R, Schneider CC, Adam P, et al. Hemangiopericytoma/solitary fibrous tumor of the greater omentum: A case report and review of the literature. Int J Surg Case Rep. 2016;23:160-2. doi: 10.1016/j.ijscr.2016.04.028.
19. Moszynski R, Szubert S, Tomczak D, et al. Solitary fibrous mass of the omentum mimicking an ovarian tumor: case report. Eur J Gynaecol Oncol. 2016;37(1):144-7.
20. Ng CS, Luqman M, Wong ZQ, et al. Gastrointestinal: Solitary fibrous tumor from lesser omentum: An elderly from Malaysia with an unusual huge painful intra-abdominal mass. J Gastroenterol Hepatol. 2017 Oct;32(10):1664. doi: 10.1111/jgh.13721.
21. Vasdeki D, Bompou E, Diamantis A, et al. Haemangiopericytoma of the greater omentum: a rare tumour requiring long-term follow-up. J Surg Case Rep. 2018 May 14;2018(5):rjy087. doi: 10.1093/jscr/rjy087.
22. Jung CY, Bae JM. Primary omental malignant solitary fibrous tumour, an extremely rare malignancy: A case report and review of the literature. Arab J Gastroenterol. 2019 Jun;20(2):114-6. doi: 10.1016/j.ajg.2018.12.001.
23. Kim S, Heo J, Kim P, et al. Solitary fibrous tumor of the lesser omentum mimicking stomach gastrointestinal stromal tumor. Korean J Clin Oncol. 2020 Dec;16(2):142-4. doi: 10.14216/kjco.20022.
24. Ingle A, Reddy S, Reddy VK. Omental Solitary Fibrous Tumor: A Rare Tumor at Rare Site. J Lab Physicians. 2021 Sep;13(3):283-5. doi: 10.1055/s-0041-1731942.

25. Eltawil KM, Whalen C, Knapp B. Solitary fibrous tumor of the greater omentum: case report and review of literature. Surg Case Rep. 2021 Apr 15;7(1):94. doi: 10.1186/s40792-021-01176-w.
26. Shahid S, Khan H, Mehmood M, et al. Malignant haemangiopericytomas of omentum presenting as left inguinal hernia: A case report. Ann Med Surg (Lond). 2021 Jan 23;62:298-301. doi: 10.1016/j.amsu.2021.01.070.
27. Guo YC, Yao LY, Tian ZS, et al. Malignant solitary fibrous tumor of the greater omentum: A case report and review of literature. World J Clin Cases. 2021 Jan 16;9(2):445-56. doi: 10.12998/wjcc.v9.i2.445.
28. Tuan HX, Hung ND, Khuong NH, et al. Primary intraperitoneal solitary fibrous tumor in mesentery: How does it present? Radiol Case Rep. 2022 Feb 18;17(4):1318-24. doi: 10.1016/j.radcr.2022.01.068.
29. Zhao M, He H, Cao D, et al. Solitary Fibrous Tumor With Extensive Epithelial Inclusions. Am J Clin Pathol. 2022 Jul 1;158(1):35-46. Erratum in: Am J Clin Pathol. 2022 Jun 7;157(6):959. doi: 10.1093/ajcp/aqab211.
30. Yin H, Ye D, Zhu Y, et al. Solitary Fibrous Tumor of the Great Omentum: A Case Report and Literature Review. Curr Med Imaging. 2022;18(4):417-20. doi: 10.2174/1573405617666211108111624.
31. Gendvilaitė N, Šeinin D, Beržanskas L, et al. Solitary Fibrous Tumor of the Peritoneal Cavity and Greater Omentum: Case Report and Review of the Literature. Acta Med Litu. 2023;30(1):66-73. doi: 10.15388/Amed.2023.30.1.7.
32. Tuan TA, Huong LM, Chau NTM, et al. Imaging Findings of Solitary Fibrous Tumors of the Gallbladder. Acta Medica (Hradec Kralove). 2024;67(3):96-100. doi: 10.14712/18059694.2025.5.
33. Hatayama R, Rino Y, Kure Y, et al. A Case of Solitary Fibrous Tumor of the Greater Omentum Incidentally Detected during Preoperative Evaluation for Thyroid Cancer. Surg Case Rep. 2025;11(1):25-0193. doi: 10.70352/scrj.cr.25-0193.
